# Genetic and Clinical Characteristics of Russian Patients with Congenital Factor V Deficiency

**DOI:** 10.3390/ijms27083646

**Published:** 2026-04-19

**Authors:** Olesya Pshenichnikova, Julia Poznyakova, Ekaterina Shchemeleva, Vadim Surin, Elena Yakovleva, Elena Likhacheva, Oksana Dimitrieva, Olga Yastrubinetskaya, Nikolay Andreev, Natalia Sats, Nadezhda Zozulya

**Affiliations:** 1 Laboratory of Genetic Engineering, National Medical Research Center for Hematology, Novy Zykovski Lane 4a, 125167 Moscow, Russia; pozniakova.i@blood.ru (J.P.); demidova.e@blood.ru (E.S.); surin.v@blood.ru (V.S.); 2 Clinical and Diagnostic Department of Hematology and Hemostasis Disorders, National Medical Research Center for Hematology, Novy Zykovski Lane 4a, 125167 Moscow, Russia; yakovleva.e@blood.ru (E.Y.); likhachyova.e@blood.ru (E.L.); dimitrieva.o@blood.ru (O.D.); yastrubinetskaya.o@blood.ru (O.Y.); andreev.n@blood.ru (N.A.); zozulya.n@blood.ru (N.Z.); 3 Laboratory of Hematopoiesis Physiology, National Medical Research Center for Hematology, Novy Zykovski Lane 4a, 125167 Moscow, Russia; sats.n@blood.ru

**Keywords:** factor V deficiency, *F5* gene, mutation spectrum, aberrant splicing, intronic variants

## Abstract

Congenital factor V (FV) deficiency is a rare autosomal recessive bleeding disorder caused by pathogenic variants in *F5* gene and characterized by heterogeneous clinical manifestations. The aim of this study was to define the mutational spectrum of *F5* in Russian patients with congenital FV deficiency. We analyzed 16 unrelated patients with different disease severity and 9 relatives from five families. All functionally relevant regions of *F5* were examined by Sanger sequencing. Multiplex ligation-dependent probe amplification (MLPA) was used to detect large deletions and duplications. Whole-genome sequencing and functional cDNA analysis were performed in selected cases. This study represents the first description of the *F5* mutational spectrum in a Russian cohort. We identified 12 novel variants and demonstrated the functional effect of two previously unreported variants located outside canonical splice-site dinucleotides, leading to aberrant splicing. Notably, the proportion of variants undetectable by routine diagnostic approaches was higher than that reported in other populations. No clear genotype–phenotype correlation was observed. Despite the limited sample size, our findings expand current knowledge of the molecular basis of congenital FV deficiency and may improve genetic diagnostics in Russia.

## 1. Introduction

Congenital factor V deficiency (OMIM #227400) is a rare autosomal recessive bleeding disorder with an estimated incidence of approximately 1 in 1,000,000 individuals. It is caused by defects in the *F5* gene, which encodes factor V (FV), also known as proaccelerin or labile factor. FV is a glycoprotein that, upon activation to FVa, functions as a cofactor for activated FX (FXa) thereby accelerating the conversion of prothrombin to thrombin. In addition to its procoagulant role, FV participates in the activated protein C-mediated inactivation of factor VIII (FVIII), thus exhibiting anticoagulant properties [1,2,3].

FV shares a structural organization similar to that of FVIII, defects of which cause hemophilia A. Both proteins contain three homologous A domains and two C domains, separated by a large B domain, and require proteolytic activation. The A and C domains of FV and FVIII share approximately 35–40% sequence homology [2,4]. FV consists of 2224 amino acids, including 28-amino acid signal peptide and a 2196-amino acid mature protein [1,2,4]. Accordingly, two nomenclature systems have been used historically; in this study, HGVS-compliant nomenclature including the signal peptide is applied throughout.

Clinically, congenital FV deficiency most commonly presents with epistaxis, easy bruising, hematomas, and menorrhagia. Severe manifestations, such as gastrointestinal bleeding, hematuria, hemarthrosis, or central nervous system hemorrhage, are relatively uncommon [2,3,5,6]. Based on residual plasma FV activity (FV:C), the disorder is generally classified as mild (FV:C ≥ 10%), moderate (FV:C < 10%), or severe (FV:C < 1%). However, a clear correlation between FV activity levels and clinical severity has not been consistently demonstrated [2,5,7].

The *F5* gene is located on chromosome 1q24.2, spans 74,680 base pairs (bp) long, and comprises 25 exons. To date, 185 pathogenic variants have been reported in the *F5* gene (HGMD, http://www.hgmd.cf.ac.uk/ac/index.php, accessed on 26 February 2026), of which 158 are associated with congenital FV deficiency. These include nonsense and frameshift variants, splice-site alterations, and large deletions; however, missense variants predominate. Most reported variants are unique and have been described in single families [2,5,7,8].

Molecular diagnosis of congenital FV deficiency is complicated by several factors. Although the disease follows an autosomal recessive inheritance pattern, both monoallelic and biallelic pathogenic variants have been reported in individuals with reduced FV activity [2,5,7,8]. In addition, functional polymorphisms—such as p.Met2148Thr and the HR2 haplotype—can reduce FV antigen levels by 25–35% even in the heterozygous state [9,10], further complicating interpretation.

Given the rarity of the disorder, its clinical heterogeneity, and the complexity of variant interpretation, expanding population-specific data on the *F5* mutational spectrum remains important. The aim of this study was to characterize the spectrum and molecular features of *F5* variants in Russian patients with congenital FV deficiency and to assess their diagnostic implications.

## 2. Results

The study sample included 16 unrelated patients with a primary diagnosis of congenital FV deficiency of different severity and 9 relatives from five families, for a total of 25 individuals (5 males and 20 females). Reduced plasma FV activity was observed in 21 individuals: five had moderate-to-severe deficiency (FV:C < 5%), and sixteen had mild deficiency (FV:C > 10%) (Table 1).

Causal *F5* variants were identified in 13 of 16 unrelated patients (81.3%) (Table 1). All five patients with FV:C < 5% carried biallelic variants: two were homozygous and three were compound heterozygotes. The remaining eight genetically supported patients carried a single heterozygous variant, although the presence of additional genetic or non-genetic modifiers cannot be excluded.

**Figure 1:**
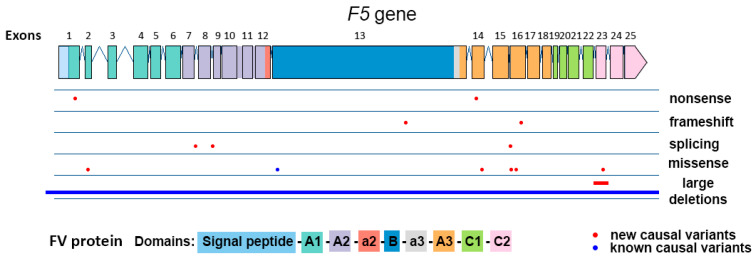
Distribution of different causal variant types found among patients with congenital FV deficiency from Russia.

In total, 15 distinct causal variants were identified, including six missense variants, three splice-site alterations, two nonsense variants, two frameshift variants, and two large deletions (Table 2). With the exception of c.1297-8C>G (detected in two unrelated patients), all variants were unique and distributed throughout the gene (Figure 1).

Only two variants had been previously reported in public databases [12,13]. Twelve were novel, and one had been described by our group earlier [11]. According to ACMG criteria, three novel missense variants (p.Glu68Lys, p.Ile1648Phe, and p.Arg2100Cys) were classified as variants of uncertain significance, two (p.Asp1739His and p.Leu1772Arg) as likely pathogenic, and the remainder as pathogenic variants (Table 2). A detailed description of all identified variants is given in Supplement Table S1.

**Figure 2:**
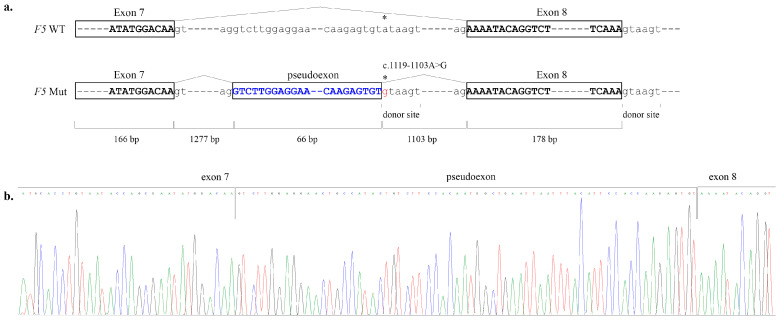
Scheme of activation of a pseudoexon by a deep intronic c.1119-1103A>G variant (**a**) (*F5* WT—normal allele, *F5* Mut—allele with alternative splicing) and (**b**) chromatogram of Sanger sequencing of *F5* cDNA with a pseudoexon of patient 12. Asterisk (*) on the scheme indicates the mutated site.

**Figure 3:**
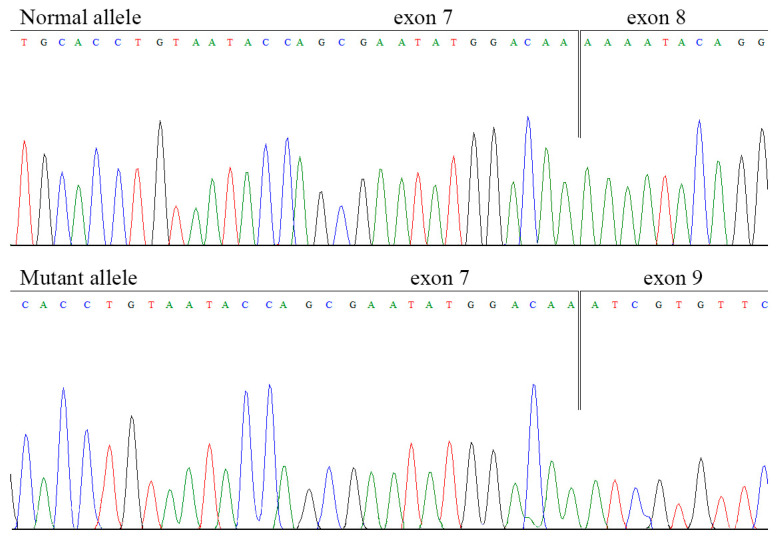
Chromatogram of Sanger sequencing of *F5* cDNA fragment of normal and mutant alleles of patient 12, demonstrating the effect of the c.1119-1103A>G variant on splicing.

In one patient (12 in Table 1), no pathogenic variants were detected by Sanger sequencing or MLPA. Whole-genome sequencing revealed a heterozygous deep intronic variant c.1119-1103A>G (rs531283306) in the *F5* gene. This variant is reported in gnomAD 4.1 at a frequency of 0.0000656; has not been described in the homozygous state and is absent from clinical variant databases. SpliceAI predicted a 0.69 probability of creating a novel donor splice site in intron 7. This site is identical to the one in exon 8 (Figure 2a). cDNA analysis confirmed aberrant splicing, but revealed two transcripts—a minor one, activating a pseudoexon in intron 7 (Figure 2) and resulting in a frameshift and truncated protein (p.Lys375SerfsTer12), and a major one without pseudoexon activation, but with exon 8 skipping, resulting in a frameshift and other truncated protein (p.Lys374SerfsTer5) (Figure 3).

Overall, 3 of 15 causal variants (20%)—including two large deletions and a non-canonical splice-altering variant—would not have been reliably detected using routine exon-focused sequencing alone.

**Figure 4:**
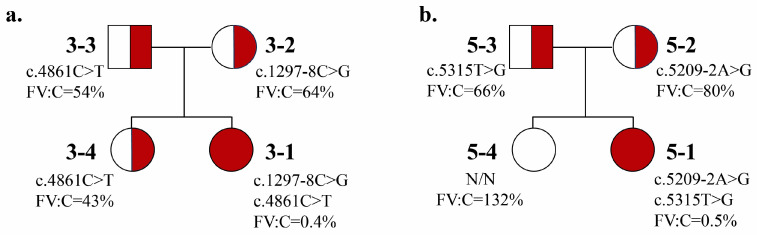
(**a**) Results of segregation analysis for the family of the patient 3; (**b**) results of segregation analysis for the family of the patient 5. The carriage of pathogenic variants and the level of FV activity for all family members are indicated.

Segregation analysis was performed in five families. In two compound heterozygous patients, the variants were confirmed to be inherited in trans (Figure 4). In patient 10-1, maternal testing showed heterozygosity for c.3913_3916del with mildly reduced FV activity (57.5%); the second variant was inherited from the father or occurred de novo. In patient 2-1, homozygous for c.1297-8C>G, the variant was confirmed to be maternally inherited. MLPA excluded deletion of the second allele; paternal material was unavailable, and parental consanguinity was denied. In patient 4-1, homozygous for c.5215G>C, the father was a heterozygous carrier. Although maternal material was unavailable, the parents were known to be first cousins, supporting autosomal recessive inheritance.

## 3. Discussion

Congenital FV deficiency is among the rarest inherited coagulation disorders. Its low prevalence and pronounced phenotypic variability have limited the availability of large population-based studies, with most published data derived from small cohorts [8,14,15,16,17,18,19,20,21] or even individual case reports [6,11,22,23,24,25,26,27,28,29,30,31,32,33,34,35,36,37,38,39,40]. Even the largest international study included only 50 patients from 11 countries [7]. In this context, the present cohort—although relatively small and clinically selected—provides additional insight into the molecular basis of the disease, while its size and potential referral bias should be considered when interpreting variant frequencies.

In our cohort, the mutational spectrum was highly heterogeneous, with predominance of unique missense variants, and no evident clear mutational hotspots (Table 2). This pattern is consistent with previous studies reports across diverse populations [7,8,14,19,21]. Founder effects have been described only in isolated populations, such as in China [16], whereas high homozygosity rates in Pakistan and Japan have been attributed to consanguinity [15,17].The high proportion of previously unreported variants in our study further supports the extensive allelic heterogeneity of *F5* [7,8,14,15,16,17,18,19,20]. This likely reflects both the large coding sequence and the rarity of the disorder, which limits accumulation of recurrent mutations in global databases [1,2].

A notable finding was the relatively high proportion of diagnostically challenging variants. Structural rearrangements and non-canonical splice-altering variants accounted for a considerable fraction of cases. The *F5* mutational spectrum appears particularly enriched for splice-disrupting variants outside canonical ±1–2 positions. Such variants—including synonymous substitutions [32], missense changes and microdeletions at exon-intron boundaries [36,41,42], variants disrupting the splicing zone [11,22,27,41,42], and deep intronic variants [21,23,41,43] have been repeatedly reported and require functional validation. While structural rearrangements are underrepresented in public databases [13,27,33], this discrepancy likely reflects methodological limitations of routine diagnostic approaches that are primarily focused on coding regions and canonical splice sites. In line with this, the *F5* gene appears to be enriched for splice-disrupting variants outside the canonical ±1–2 positions, including synonymous substitutions, missense changes affecting exon–intron boundaries, and deep intronic variants. These mechanisms require transcript-level or functional validation and may be overlooked by standard exon-focused sequencing strategies. Compared with other coagulation genes such as *F8* [44], these features may reflect differences in genomic organization and regulatory architecture.

A key limitation of this study is the limited functional validation of identified variants. RNA analysis confirmed the splicing effect of one deep intronic variant and prior work from our group has demonstrated the impact of another non-canonical splice-site variant [11]. However, most novel missense variants were interpreted based on ACMG/AMP criteria, including in silico prediction, population frequency, and segregation data where available. Therefore, their pathogenicity should be regarded as inferred rather than experimentally confirmed. Additional functional studies, including expression assays and protein characterization, are needed to better define their impact on FV synthesis, secretion, and activity.

The inclusion of patients with a wide range of FV activity allowed assessment of genotype–phenotype relationships across the clinical spectrum. Consistent with previous studies [7,8,14,15,18,19], no strict genotype–phenotype correlation was observed. Although lower FV activity was generally associated with more pronounced bleeding, individual variability was substantial, ranging from asymptomatic disease (6 individuals) to a history of severe gastrointestinal bleeding or cephalohematoma (5 individuals). Notably, severe bleeding occurred in an individual with FV:C of 40%, whereas another patient with FV:C of 4% presented only with mild mucosal bleeding. Data on the ISTH-BAT was missing for only one of the relatives. The average value for the entire sample was 7.1 points. For individuals with identified genetic variants in the *F5* gene, the average value was 8 points. For homozygotes and compound heterozygotes (N = 5), the average value was 21.6 points, and among carriers of one heterozygous variant (N = 15), it was 3.5 (Table 1).

This variability likely reflects a combination of biological and methodological factors. From a biological perspective, the dual origin of FV from plasma and platelet pools may partially compensate for reduced circulating levels [1,2]. In addition, variability in other components of the coagulation system and the presence of genetic modifiers may influence the clinical phenotype. Variants associated with the HR2 haplotype and p.Met2148Thr have been reported to modulate FV levels [9,10], and co-inherited prothrombotic or bleeding disorders may further contribute to phenotypic diversity [8]. Non-genetic factors, including environmental influences and comorbid conditions, may also affect FV activity and bleeding risk. From a methodological perspective, the relatively small sample size and heterogeneity in clinical presentation may limit the ability to detect consistent associations between genotype and clinical severity. Although bleeding severity was assessed using a standardized bleeding assessment scale, some variability in phenotypic expression is inherent to this condition.

Interpretation of monoallelic variants in FV deficiency remains particularly challenging. In our cohort, heterozygous variants were generally associated with moderately reduced FV levels, consistent with previous observations [2,5,7,8]. However, undetected variants, modifier alleles, or complex genetic backgrounds may contribute to the phenotype in such cases.

Causal variants were identified in approximately 80% of unrelated patients, in line with previous studies reports indicating that 10–20% of individuals with reduced FV activity lack clearly pathogenic variants [7,8,14,27]. In the remaining cases, functional polymorphisms or combinations of low-impact variants may explain reduced FV activity. Specific substitutions within HR2 (including p.Met413Thr, p.His1327Arg, p.Met1764Val, and p.Asp2222Gly, or according to the legacy nomenclature: p.Met385Thr, p.His1299Arg, p.Met1736Val and p.Asp2194Gly) have been previously shown to reduce FV antigen levels by 25–35% even in the heterozygous state, similar to p.Met2148Thr [9,10]. In our cohort, two patients carried the HR2 haplotype, and one carried both HR2 and p.Met2148Thr. Similar mechanisms may contribute to phenotypic variability in our cohort, although this was not functionally assessed. Epigenetic mechanisms, such as DNA methylation in regulatory regions, may also influence *F5* gene expression and represent an additional layer of regulation that was not addressed in the present study.

Taken together, our findings indicate that the mutational landscape of *F5* is characterized not only by marked allelic heterogeneity but also by a significant proportion of variants that are not readily detectable using standard diagnostic approaches. This has important implications for molecular diagnostics. A stepwise strategy appears to be most effective. Initial testing should focus on exon-targeted sequencing using Sanger methods or targeted NGS panels, which capture the majority of pathogenic variants and allow exclusion of alternative genetic causes of the phenotype. In patients without a confirmed diagnosis, copy number analysis should be performed to detect structural variants. In unresolved cases, particularly those with strong clinical suspicion, more comprehensive approaches such as whole-genome sequencing or RNA analysis may be required to identify deep intronic or splicing defects. From a practical perspective, first-line sequencing approaches are widely available, relatively cost-effective, and have shorter turnaround times, making them suitable for routine diagnostics. In contrast, MLPA and especially WGS or RNA-based studies are more resource-intensive and less universally available, but can provide critical diagnostic yield in selected cases. Such a tiered approach allows optimization of diagnostic yield while balancing cost and resource availability.

From a clinical perspective, identification of variants in the *F5* gene has important implications for genetic counseling. Molecular diagnosis enable accurate assessment of carrier status and recurrence risk within affected families. However, the absence of a clear genotype–phenotype correlation limits the predictive value of genetic findings for clinical severity. This should be taken into account when interpreting results, particularly in the context of reproductive decision-making. Prenatal or preimplantation genetic testing should therefore be considered on an individual basis within comprehensive genetic counseling.

Although clinical management was not addressed in this study, knowledge of the underlying genetic defect may support risk assessment in specific situations, such as surgery or pregnancy. Overall, the primary clinical value of *F5* genotyping lies in improving diagnostic accuracy and supporting informed decision-making in patients and their families.

## 4. Materials and Methods

During the preparation of this work the authors did not use generative AI or AI-assisted technologies.

The study included data from 16 unrelated patients with a primary diagnosis of congenital FV deficiency of different severity and 9 relatives from five families, after obtaining written informed consent. The data were collected between 2016 and 2025 from patients from different regions of Russia, who were referred to the National Medical Research Center for Hematology. This may result in enrichment for clinically more severe or diagnostically challenging case, but this center serves as a national referral center for adult patients with suspected congenital FV deficiency, enabling access to genetically confirmed cases. All procedures performed in the study involving human subjects complied with the ethical standards of the institutional and/or national research ethics committee and the 1964 Helsinki Declaration and its later amendments, or comparable ethical standards.

Congenital FV deficiency was defined based on isolated, persistently reduced FV activity (<70%) measured on at least two independent occasions. Bleeding severity was assessed using the ISTH Bleeding Assessment Tool (ISTH-BAT), where available. Scores were used to standardize the evaluation of hemorrhagic manifestations across patients.

Acquired causes of FV deficiency (including liver disease, anticoagulant therapy, and inhibitors) were excluded based on comprehensive clinical and laboratory evaluation, including assessment of liver function (albumin, bilirubin, AST/ALT), extended coagulation testing, dilute Russell viper venom time, testing for FV inhibitors, and liver ultrasound examination. FV activity measurements were performed outside of acute bleeding episodes, pregnancy, and ongoing therapy. Laboratory testing was conducted under stable clinical conditions. None of the patients received anticoagulant therapy at the time of evaluation.

No patients meeting these criteria were excluded from the study, including individuals with mild FV deficiency.

Genomic DNA was isolated from EDTA-treated whole blood samples using phenol–chloroform extraction and ethanol precipitation, dissolved in TE buffer and frozen until genotyping [45].

All 25 exons, exon-intron junctions, and the promoter region (approximately 600 bp upstream of the transcription start site) of the *F5* gene were systematically amplified and analyzed by Sanger sequencing in all patients. Amplification was performed using the PCR Master Mix system (Thermo Fisher Scientific, Waltham, MA, USA) with 0.01–0.02 μg of genomic DNA at 94 °C for 1 min, 62 °C for 1 min, and 72 °C for 3 min for 30 cycles. The longest exon, exon 13, was amplified using the long-distance PCR (LD-PCR) technique using the Promega GoTaq^®^Long PCR Master Mix (Promega Corporation, Madison, WI, USA). PCR products were visualized by electrophoresis. All primers used in the work [11] were synthesized at Syntol (Syntol, Moscow, Russia).

PCR fragments for sequencing were purified on Wizard columns (Promega Corporation). Sanger sequencing was performed using the BrilliantDye Terminator v.1.1 reagent kit (NimaGen, Nijmegen, the Netherlands), followed by analysis of the reaction products on a Nanofor 05 automated sequencer (Syntol).

Large deletions/insertions were tested with multiplex ligation-dependent probe amplification (MLPA) in all 16 unrelated patients with a primary diagnosis of congenital FV deficiency. MLPA was carried out using the F5 SALSA MLPA kit P469 and SALSA MLPA Reagent Kit (MRC Holland, Amsterdam, The Netherlands) following the manufacturer’s protocol. Exon dosage was calculated using Coffalyser.Net v.240129.1959 software (MRC Holland). Relative peak areas were used to determine the dosage quotient (DQ) for each probe. Standard threshold values were applied for clinical interpretation: a DQ between 0.7 and 1.3 was considered normal (diploid), while values below 0.7 or above 1.3 were indicative of heterozygous deletions or duplications, respectively.

Whole-genome sequencing (WGS) was conducted in four cases with unresolved genotype after routine testing (patients 7, 12, 13 and 16) in collaboration with Biotechnology Campus LLC as part of the national genetic initiative “100,000+ Me.” It was performed using the PE150 paired-end read method on a DNBSEQ-T7 genetic analyzer (MGI Tech Co., Shenzhen, China). Reads were aligned with bwa-mem tool using hg38 (GRCh38) as a reference. Variants were called with DeepVariant caller and Manta Structural Variant Caller and annotated with Open-CRAVAT python package 2.17.0. The mean coverage exceeded 40×, with a minimum depth threshold of 10× and Phred Q30 for variant calling. Our study specifically focused on the *F5* gene as the primary cause of the phenotype, so the filtering strategy was centered on identifying rare variants (MAF < 0.01 in gnomAD) within the *F5* locus. Only variants located within the *F5* gene were considered for further analysis in accordance with the study objectives. Variants revealed by WGS were verified by Sanger sequencing.

The identified variants were named according to the HGVS (Human Genome Variation Society, http://varnomen.hgvs.org/, accessed on 27 February 2026) recommendations, using DNA (NG_011806.1) and mRNA (NM_000130.5) sequences as reference. The identified variants were checked against databases (https://dbs.eahad.org/FV, accessed on 27 February 2026; http://www.hgmd.cf.ac.uk/, accessed on 27 February 2026; https://www.ncbi.nlm.nih.gov/clinvar/, accessed on 27 February 2026), and previously undescribed variants were tested for pathogenicity using the ACMG/AMP Variant Curation Guidelines [46] and its updates. Multiple lines of evidence were integrated, including population frequency data (gnomAD), computational predictions (including splice prediction tools where applicable), segregation analysis (when available), previously reported cases, and functional data. When potentially conflicting evidence was present (e.g., variants of uncertain significance with limited supportive segregation data), each criterion was weighted according to ACMG/AMP recommendations. Segregation data were considered as supporting evidence only when consistent with the expected inheritance pattern and sufficient family data were available, but were not used to upgrade variant classification in the absence of additional supporting evidence. Final variant classification (pathogenic, likely pathogenic, VUS, likely benign, benign) was assigned based on the overall combination of criteria. Detailed evidence for each variant is provided in Supplement Table S1.

To determine the potential impact of the identified deep intronic variant on splicing, in silico analysis was performed using the SpliceAI predictive algorithm [47].

To assess the functional impact of the deep intron variant, *F5* cDNA was sequenced using total RNA samples isolated from the patient’s and healthy donor’s nuclear cells as a template using the RNA-Extran kit (Syntol). Reverse transcription was performed using the OT-1 kit (Syntol) and the F5R primer (5′-GGTGTTGTTCCTGCCTGAGGTGA-3′). The resulting *F5* cDNA fragment, covering part of exon 6, the entirety of exons 7 and 8, and part of exon 9, was amplified by nested PCR under standard conditions in the PCR Master Mix system (Thermo Fisher Scientific): the first amplification step was performed using primers F5RT6D (5′-CTCTCACCCCAAAACATTTGCA-3′) and F5R (5′-GGTGTTGTTCCTGCCTGAGGTGA-3′), the second step was performed with the PCR product obtained as a result of the first step and diluted 1:100, with primers F5RT6D and F5R9RT (5′-GCGAGAAGGTCACTCCATGA-3′). To get a minor transcript another step of nested PCR was conducted: with primers F5D7RT (5′-GGACTATGCACCTGTA-3′) and F5R8RT (5′-GGACCCAAAATCCCATCTTCT-3′). Sanger sequencing was performed as described above.

## 5. Conclusions

This study represents the first comprehensive analysis of the genetic characteristics of patients with congenital factor V deficiency in Russia. Overall, the mutational spectrum of the *F5* gene in our cohort is consistent with global observations, including the presence of both monoallelic and biallelic variants, a high proportion of private (family-specific) mutations, and their relatively uniform distribution across the gene.

Notably, we observed a comparatively high proportion of variants that are unlikely to be detected by routine exon-focused diagnostic approaches, including large deletions and non-canonical splice-altering variants. Although this finding should be interpreted with caution given the limited cohort size, it underscores the importance of incorporating structural variant analysis and transcript-level studies into the diagnostic workflow for congenital FV deficiency.

While missense variants predominated in our cohort, their interpretation remains challenging due to the high background frequency of benign and functional nonsynonymous polymorphisms in *F5*. In this context, segregation analysis within families proved particularly valuable for clarifying variant pathogenicity and refining clinical interpretation.

Despite the relatively small sample size, our findings expand the current understanding of the molecular architecture of congenital FV deficiency and highlight key considerations for optimizing genetic diagnostics in rare coagulation disorders.

## Figures and Tables

**Table 1:** Description of the patient sample and the causal variants identified in the *F5* gene.

Family	Patient	Sex	FV, % (Normal Range Is 70–120)	Clinical Manifestations	ISTH-BAT	Causal Variant (HGVS)	Zygosity	Polymorphisms *
1	1	F	24	In childhood, ecchymosis, epistaxis, bleeding after tooth extraction, menorrhagia	6	c.2051G>A (p.Cys684Tyr)	hetero	-
2	2-1	M	0.5	Hemarthrosis, hematoma, gastrointestinal bleeding, ecchymosis, hematuria	18	c.1297-8C>G (p.Ile433PhefsTer13)	homo	-
2	2-2 (mother)	F	78.8	No	0	c.1297-8C>G (p.Ile433PhefsTer13)	hetero	-
3	3-1	F	0.4	Ecchymosis, epistaxis, post-traumatic bleeding and hematomas, menometrorrhagia, gastrointestinal bleeding, recurrent hematuria, bleeding after tooth extraction, bleeding after surgery	35	c.1297-8C>G (p.Ile433PhefsTer13)	hetero	
						c.4861C>T (p.Arg1621Ter)	hetero	
3	3-2 (mother)	F	64.0	Menometrorrhagia	3	c.1297-8C>G (p.Ile433PhefsTer13)	hetero	
3	3-3 (father)	M	54.0	No	0	c.4861C>T (p.Arg1621Ter)	hetero	
3	3-4 (sibling)	F	43.0	Long-term healing of postoperative wounds, ecchymosis	4	c.4861C>T (p.Arg1621Ter)	hetero	
4	4-1	F	4	Gum bleedings, bleeding after surgery	5	c.5215G>C (p.Asp1739His)	homo	
4	4-2 (father)	M	ND **	ND	ND	c.5215G>C (p.Asp1739His)	hetero	
5	5-1	F	0.5	Cephalohematoma, hematomas, hemarthrosis, ecchymosis, epistaxis, gum bleedings, retroperitoneal hematoma, menorrhagia, bleeding during teeth change.	28	c.5209-2A>G	hetero	
						c.5315T>G (p.Leu1772Arg)	hetero	
5	5-2 (mother)	F	79.7	No	0	c.5209-2A>G	hetero	
5	5-3 (father)	M	66.3	No	0	c.5315T>G (p.Leu1772Arg)	hetero	
5	5-4 (sibling)	F	132	No	0	-	-	
6	6	F	47.4	Menometrorrhagia, epistaxis, gum bleedings	4	c.6298C>T (p.Arg2100Cys)	hetero	p.Met2148Thr hetero
7	7	F	11	Prolonged bleeding after tooth extraction, gum bleedings, pre-pregnancy menorrhagia	5	-	-	HR2 hetero
8	8	F	40	Epistaxis, ecchymosis, gastrointestinal bleeding, bleeding after surgery	10	c.202G>A (p.Glu68Lys)	hetero	p.Met2148Thr hetero
9	9	F	63	Ecchymosis (mainly on legs)	2	c.4942A>T (p.Ile1648Phe)	hetero	
10	10-1	F	0.6	Cephalohematoma, severe epistaxis and gum bleedings, ecchymosis, prolonged post-traumatic bleeding and bleeding after blood sampling, menorrhagia, apoplexy of both ovaries, intra-abdominal bleeding, bleeding after tooth extraction	22	c.97C>T (p.Gln33Ter)	hetero	
						c.3913_3916del	hetero	
10	10-2 (mother)	F	57.5	Bleeding after surgery, postpartum hemorrhage	3	c.3913_3916del	hetero	
11	11	F	44.2	Ecchymosis, post-traumatic hematomas, prolonged bleeding after cuts, epistaxis and provocative gum bleeding	9	c.5357dup (p.Glu1785GlufsTer19)	hetero	
12	12	F	42.6	Epistaxis, provocative gum bleeding, postoperative bleeding, prolonged bleeding after cuts, ecchymosis, menorrhagia	8	c.1119-1103A>G (p.Lys374SerfsTer5)	hetero	p.Met2148Thr hetero
13	13	M	56.1	Petechiae, ecchymosis, prolonged bleeding after tooth extraction	2	-	-	p.Met2148Thr hetero; HR2 hetero
14	14	F	33.5	No	0	exon 23 del	hetero	HR2 hetero
15	15	F	28.1	Intense epistaxis, ecchymosis	4	LRG_553: g.(?_167748821)_(167822136_?)del (delexon1-25)	hetero	p.Met2148Thr homo
16	16	F	45	Epistaxis, menorrhagia	2	-	-	HR2 hetero

* Functional polymorphisms p.Met2148Thr and HR2. ** No data.

**Table 2:** Description of identified causal variants in the *F5* gene.

No.	Causal Variant (HGVS)	Exon/Intron	Domain	Type	Reference	Pathogenicity of Novel Variant According to ACMG
1	c.97C>T (p.Gln33Ter)	Exon 1	A1	Nonsense	new	Pathogenic (PM2, PVS1, PM3)
2	c.4861C>T (p.Arg1621Ter)	Exon 14	A3	Nonsense	new	Pathogenic (PM2, PVS1, PP1, PM3, PP4)
3	c.3913_3916del (p.Pro1305AsnfsTer27)	Exon 13	B	Frameshift	new	Pathogenic (PM2, PVS1, PP1, PM3)
4	c.5357dup (p.Glu1785GlufsTer19)	Exon 16	A3	Frameshift	new	Pathogenic (PM2, PVS1, PP4)
5	c.1119-1103A>G (p.Lys374SerfsTer5)	Intron 7	A2	Splicing	new	Pathogenic (PM2, PVS1, PS3, PP4)
6	c.1297-8C>G (p.Ile433PhefsTer13)	Intron 8	A2	Splicing	[11]	Pathogenic (PM2, PVS1, PS3, PP4)
7	c.5209-2A>G	Intron 15	A3	Splicing	new	Pathogenic (PM2, PVS1, PP1, PP4)
8	c.202G>A (p.Glu68Lys)	Exon 2	A1	Missense	new	VUS * (PM2, PP3, PP4)
9	c.2051G>A (p.Cys684Tyr)	Exon 13	A2	Missense	[12]	
10	c.4942A>T (p.Ile1648Phe)	Exon 14	A3	Missense	new	VUS (PM2, PP3, PP4)
11	c.5215G>C (p.Asp1739His)	Exon 16	A3	Missense	new	Likely pathogenic (PM2, PM5, PP3, PP4)
12	c.5315T>G (p.Leu1772Arg)	Exon 16	A3	Missense	new	Likely pathogenic (PM2, PP3, PP1, PM3, PP4)
13	c.6298C>T (p.Arg2100Cys)	Exon 23	C2	Missense	new	VUS (PM2, PP3, PP4)
14	exon 23 del	Exon 23	C2	Large deletion	new	Pathogenic (PM2, PVS1, PP4)
15	LRG_553: g.(?_167748821)_(167822136_?)del (delexon1-25)	Exon 1-25	A1-C2	Large deletion	[13]	

* VUS—variant of uncertain significance.

## Data Availability

The original contributions presented in this study are included in the article. Further inquiries can be directed to the corresponding author.

## Supplementary Materials

The following supporting information can be downloaded at: https://www.mdpi.com/article/10.3390/ijms27083646/s1.

## Abbreviations

The following abbreviations are used in this manuscript:

HGVS	Human Genome Variation Society (nomenclature system)
HGMD	Human Gene Mutation Database
ACMG	American College of Medical Genetics and Genomics
cDNA	Complementary DNA
MLPA	Multiplex ligation-dependent probe amplification
gnomAD	Genome Aggregation Database
WGS	Whole genome sequencing
DNA	Deoxyribonucleic acid
RNA	Ribonucleic acid
mRNA	Matrix ribonucleic acid
EDTA	Ethylenediaminetetraacetic acid
PCR	Polymerase chain reaction
LD-PCR	Long-distance polymerase chain reaction
bp	Base pairs
VUS	Variant of uncertain significance
